# Supplementary material for: Design, Synthesis and Phenotypic Profiling of Simplified Gedatolisib Analogues
Source: Pharmaceuticals (Basel). 2023 Jan 30;16(2):209. doi: 10.3390/ph16020209 (PMC9964390; doi:10.3390/ph16020209)
Supplement: Supplementary file 1 [file pharmaceuticals-16-00209-s001.zip › pharmaceuticals-2164508-supplementary.pdf]

Supplementary Materials

DESIGN, SYNTHESIS AND PHENOTYPIC PROFILING OF SIMPLIFIED GEDATOLISIB ANALOGUES

Caroline Marques Xavier Costa[1,3], Cristiane Aparecida-Silva[1,3], Luis Eduardo Reina Gamba[1,3], Thalita Neves de Melo[1], Gisele Barbosa[1,3], Manoel Oliveira de Moraes Junior[1,3], Victoria Regina Thomaz de Oliveira[1,3], João Alfredo de Moraes[2], Carolinne Souza de Amorim[2], Eliezer J. Barreiro[1,3], Lídia Moreira Lima[1,3].

[1]*Instituto Nacional de Ciência e Tecnologia de Fármacos e Medicamentos (INCT-INOFAR), Universidade Federal do Rio de Janeiro, Laboratório de Avaliação e Síntese de Substâncias Bioativas (LASSBio®), CCS, Cidade Universitária, P.O. Box 68024, 21941-971, Rio de Janeiro, RJ, Brasil.*

[2]*Laboratório de Biologia Redox (LABIO-RedOx®), Instituto de Ciências Biológicas, Universidade Federal do Rio de Janeiro, RJ, Brasil.*

[3]*Programa de Pós-graduação em Farmacologia e Química Medicinal, Instituto de Ciências Biomédicas, Universidade Federal do Rio de Janeiro, Rio de Janeiro, RJ, Brasil*

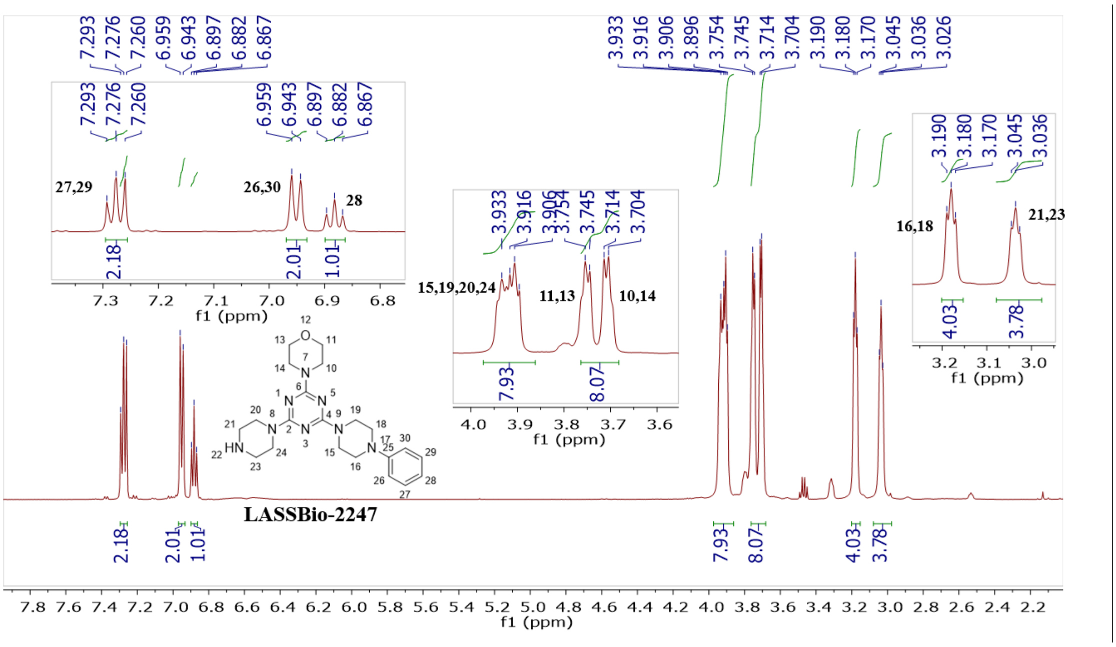

Figure S1. ^{1}H NMR spectra of 4-(4-(4-phenylpiperazin-1-yl)-6-(piperidin-1-yl)-1,3,5-triazin-2-yl)morpholine (**5c**) (400 MHz, DMSO-d_6).

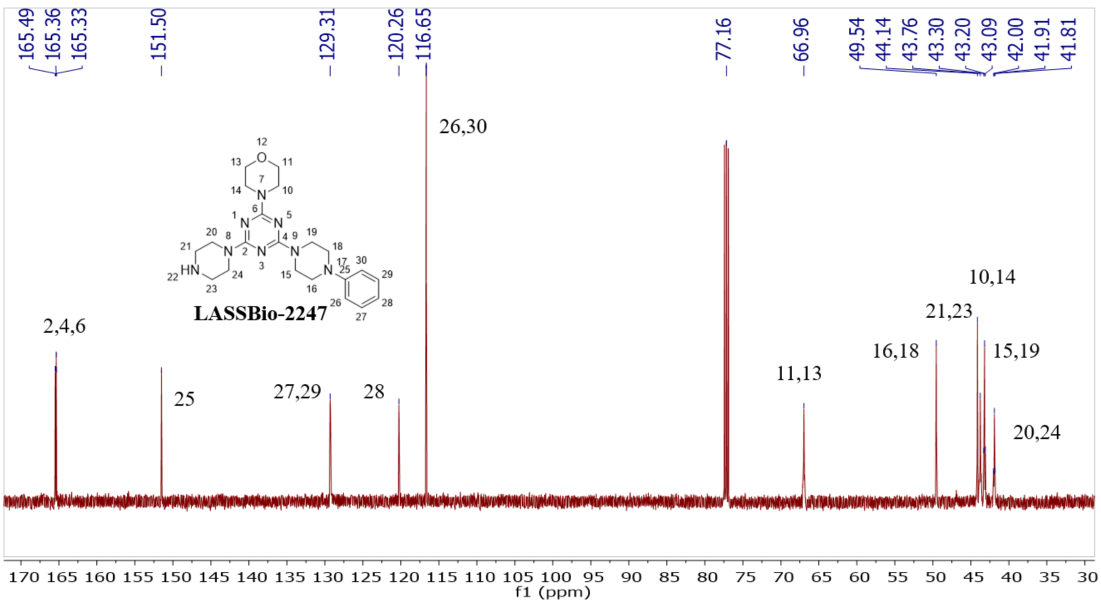

Figure S2. ^{13}C NMR spectra of 4-(4-(4-phenylpiperazin-1-yl)-6-(piperidin-1-yl)-1,3,5-triazin-2-yl)morpholine

(5**c**) (400 MHz, $CDCl_3$).

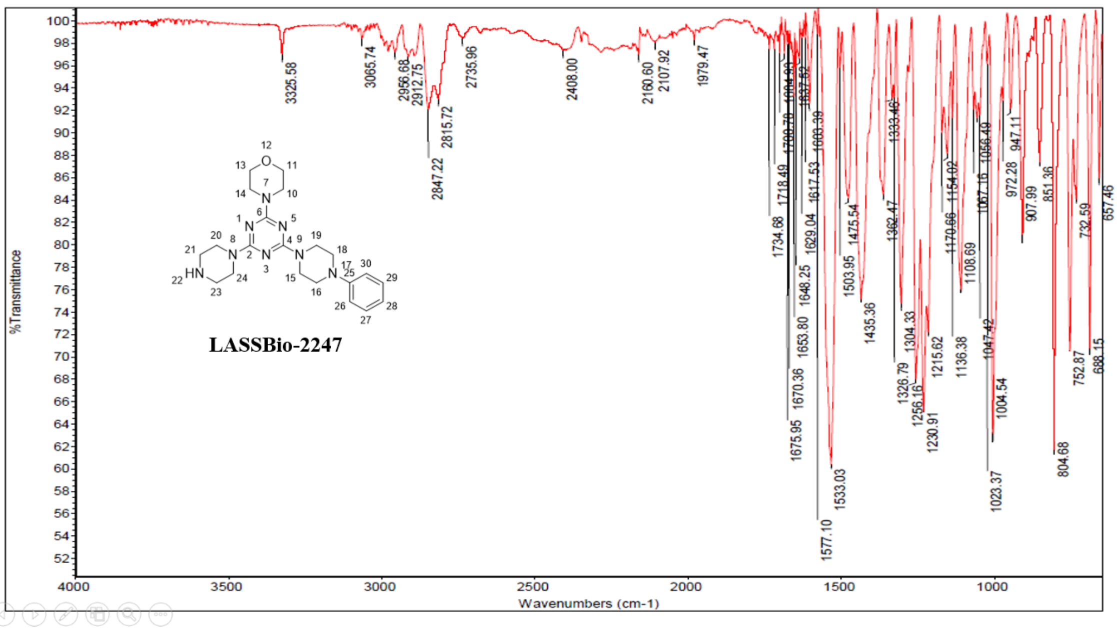

Figure S3. Absorption spectra in the infrared region of 4-(4-(4-phenylpiperazin-1-yl)-6-(piperidin-1-yl)-1,3,5-triazin-2-yl)morpholine (5**c**) (ATR).

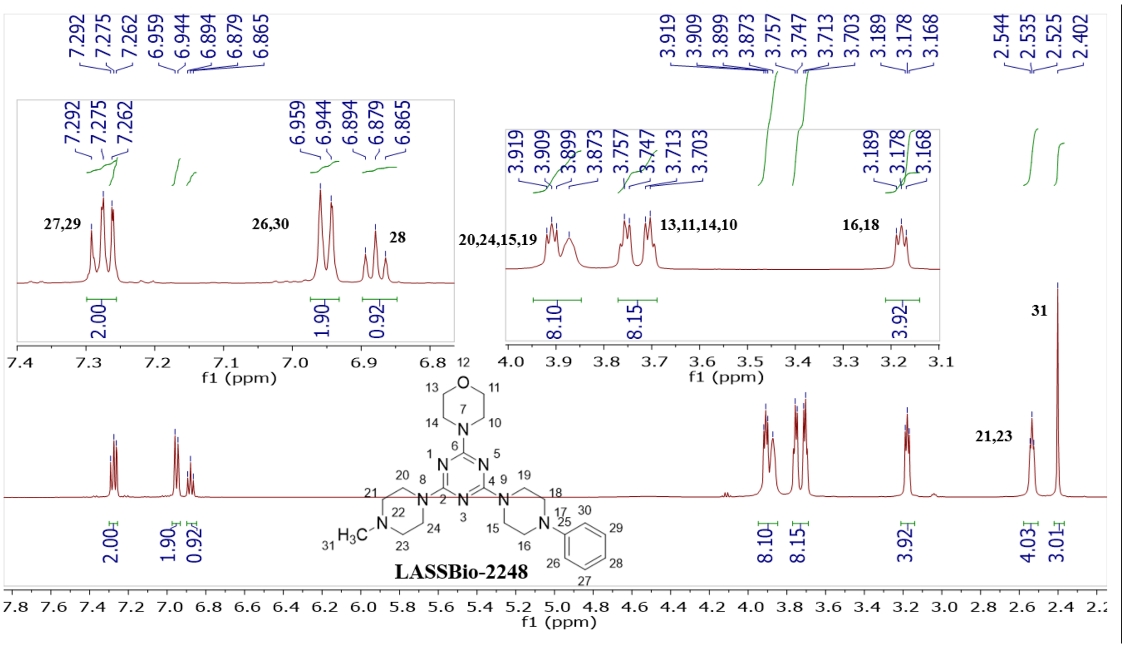

Figure S4. ^{1}H NMR spectra of 4-(4-(4-methylpiperazin-1-yl)-6-(4-phenylpiperazin-1-yl)-1,3,5-triazin-2-yl)morpholine (**5d**) (400 MHz, DMSO-*d*6).

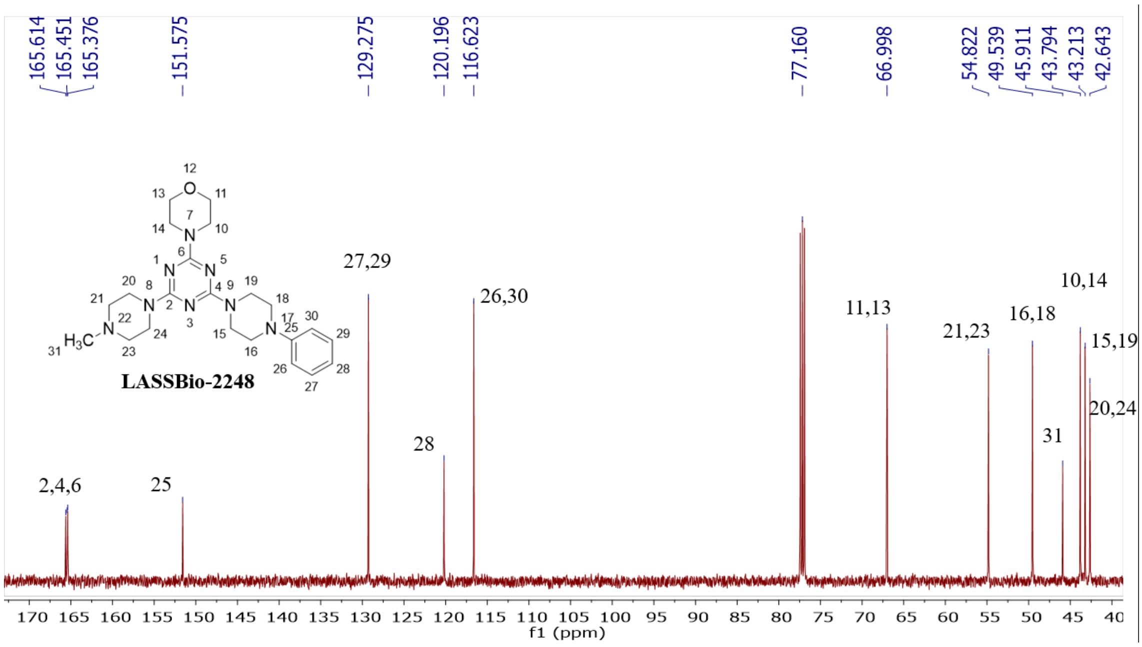

Figure S5. ^{13}C NMR spectra of 4-(4-(4-methylpiperazin-1-yl)-6-(4-phenylpiperazin-1-yl)-1,3,5-triazin-2-yl)morpholine (**5d**) (400 MHz, CDCl3).

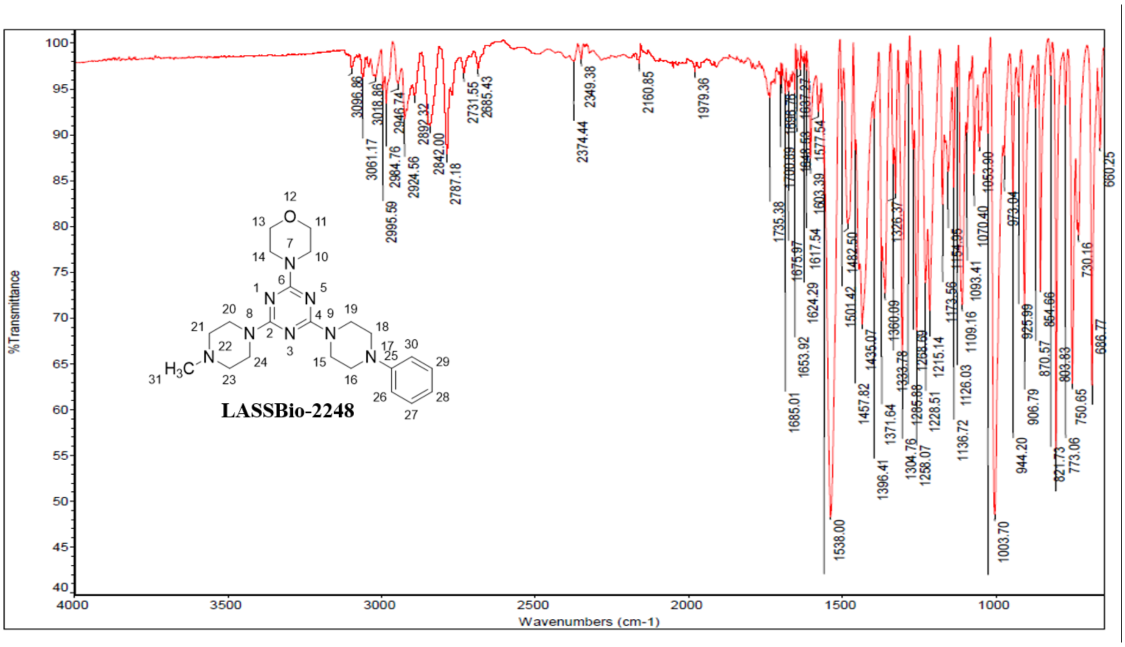

Figure S6. Absorption spectra in the infrared region of 4-(4-(4-methylpiperazin-1-yl)-6-(4-phenylpiperazin-1-yl)-1,3,5-triazin-2-yl)morpholine (**5d**) (ATR).

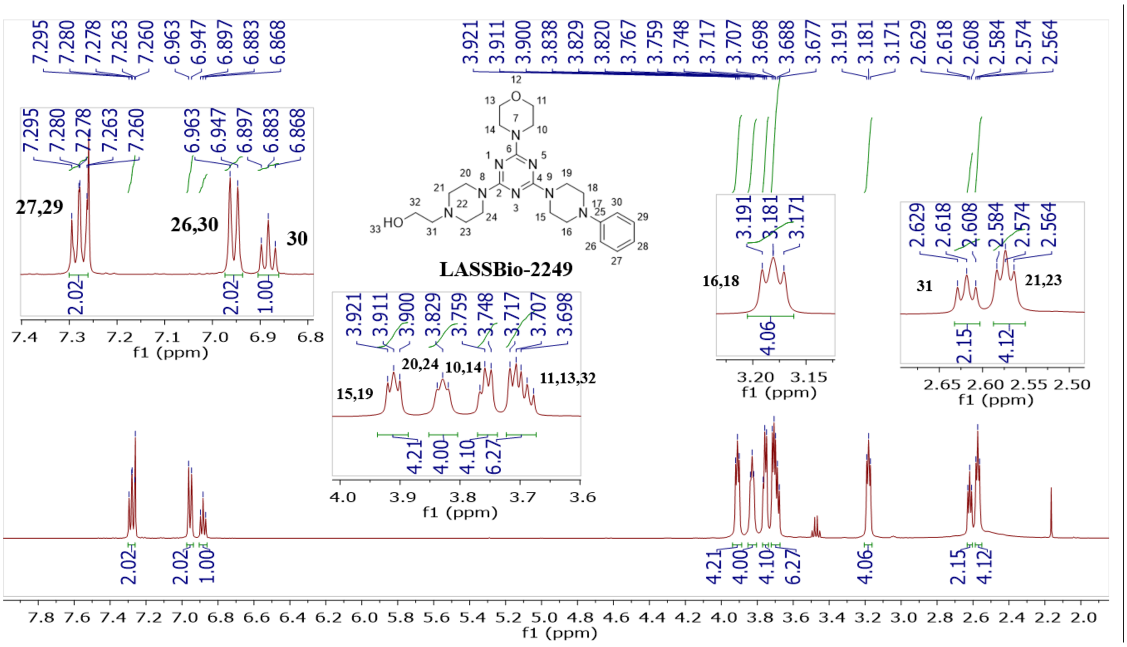

Figure S7. ^{1}H NMR spectra of 2-(4-(4-morpholino-6-(4-phenylpiperazin-1-yl)-1,3,5-triazin-2-yl)piperazin-1-yl)ethan-1-ol (**5e**) (400 MHz, DMSO-d_6).

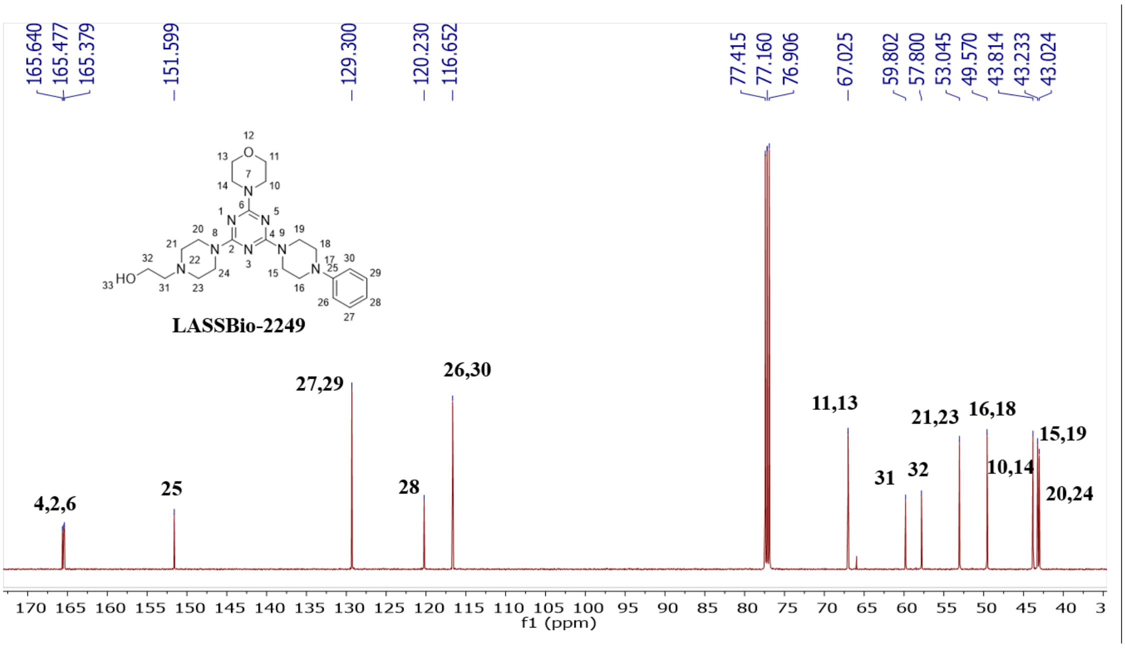

Figure S8. ^{13}C NMR spectra of 2-(4-(4-morpholino-6-(4-phenylpiperazin-1-yl)-1,3,5-triazin-2-yl)piperazin-1-yl)ethan-1-ol (**5e**) (400 MHz, $CDCl_3$).

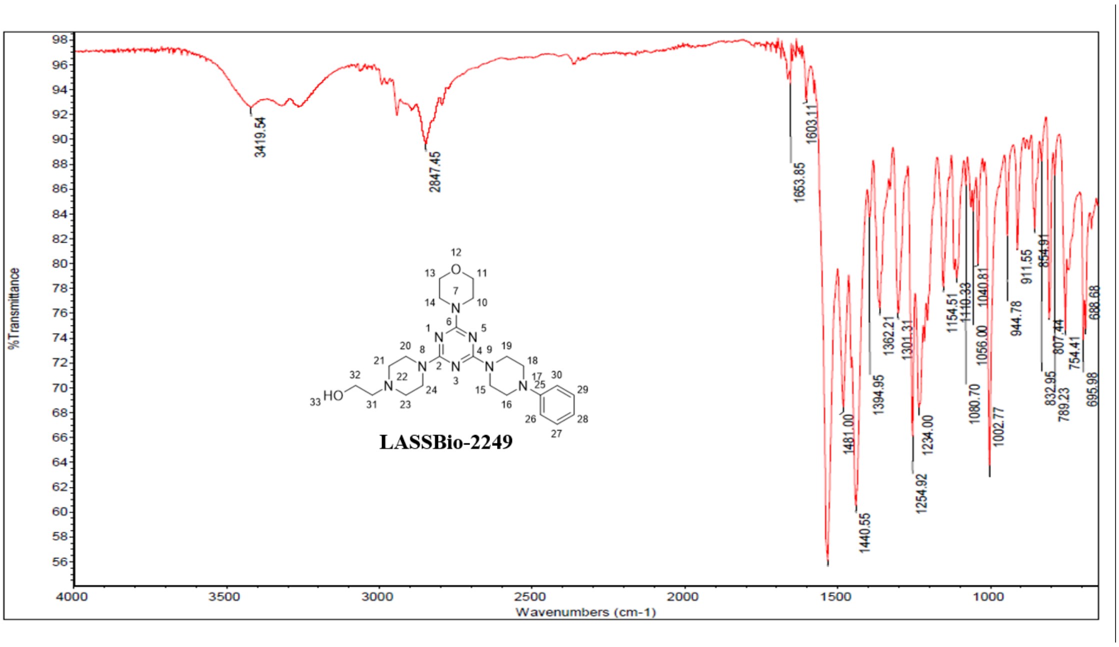

Figure S9. Absorption spectra in the infrared region of 2-(4-(4-morpholino-6-(4-phenylpiperazin-1-yl)-1,3,5-triazin-2-yl)piperazin-1-yl)ethan-1-ol (**5e**) (ATR).

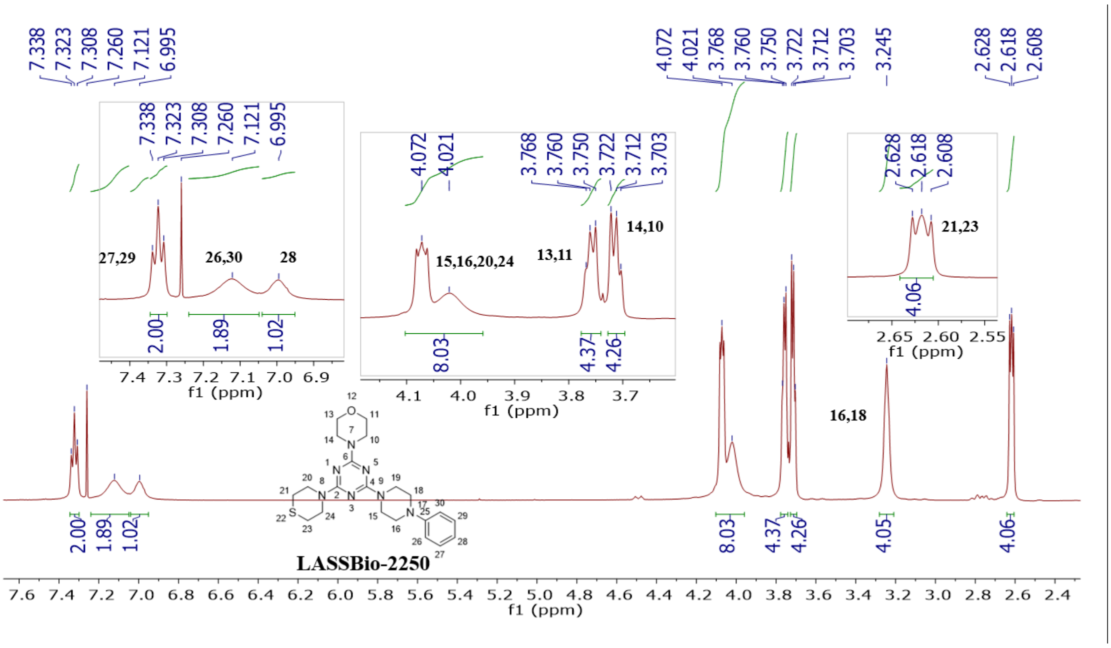

Figure S10. ^{1}H NMR spectra of 4-(4-(4-phenylpiperazin-1-yl)-6-thiomorpholino-1,3,5-triazin-2-yl)morpholine (**5a**) (400 MHz, DMSO-d_6).

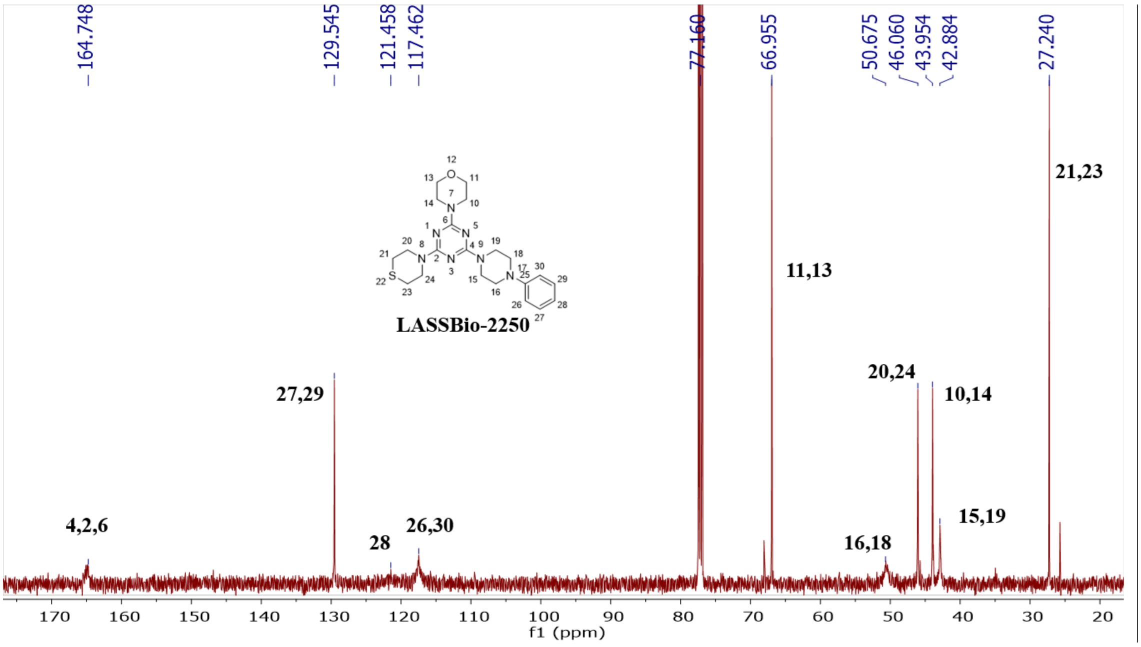

Figure S11. ^{13}C NMR spectra of 4-(4-(4-phenylpiperazin-1-yl)-6-thiomorpholino-1,3,5-triazin-2-yl)morpholine

(**5a**) (400 MHz, $CDCl_3$).

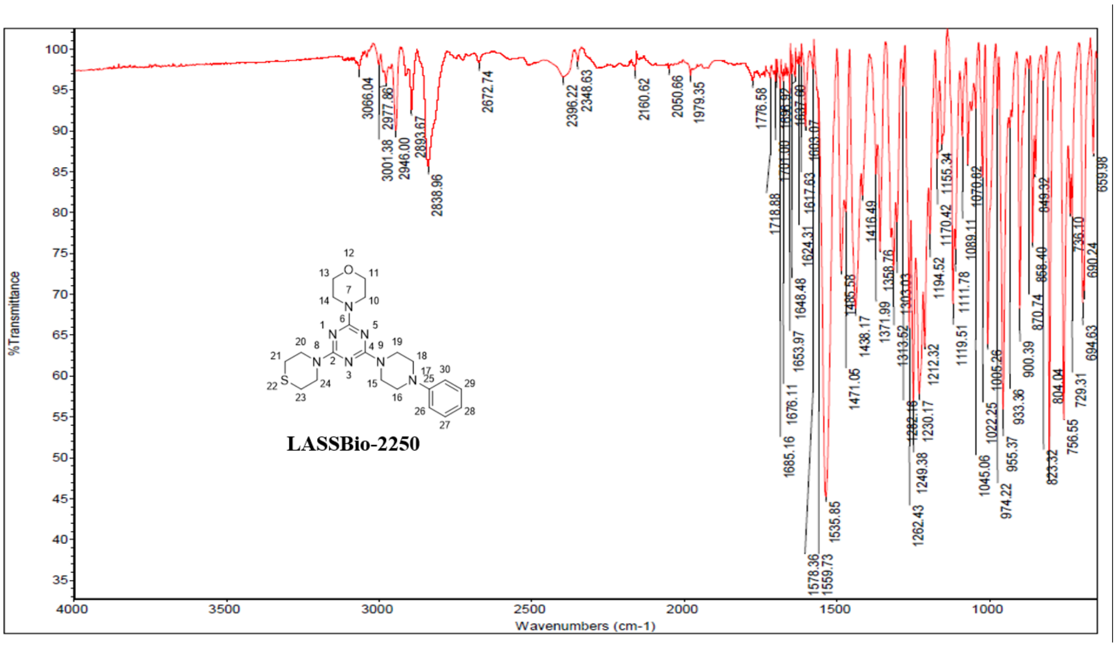

Figure S12. Absorption spectra in the infrared region of 4-(4-(4-phenylpiperazin-1-yl)-6-thiomorpholino-1,3,5-triazin-2-yl)morpholine (**5a**) (ATR).

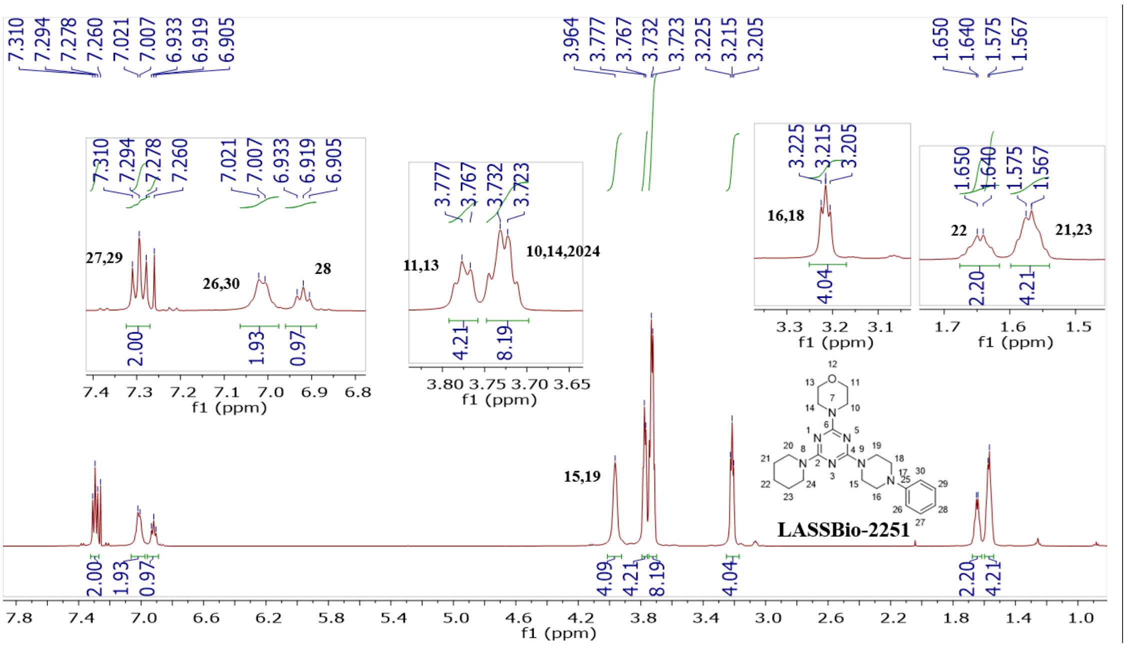

Figure S13. ^{1}H NMR spectra of 4-(4-(4-phenylpiperazin-1-yl)-6-(piperidin-1-yl)-1,3,5-triazin-2-yl)morpholine (**5b**) (400 MHz, DMSO-d_6).

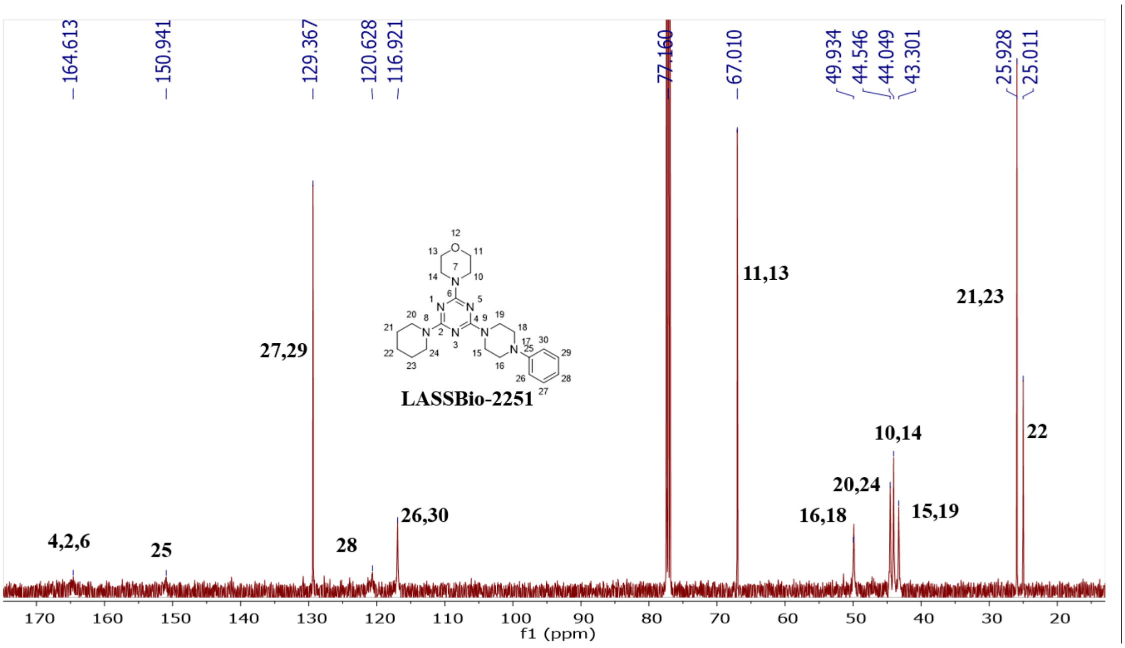

Figure S14. ^{13}C NMR spectra of 4-(4-(4-phenylpiperazin-1-yl)-6-(piperidin-1-yl)-1,3,5-triazin-2-yl)morpholine (**5b**) (400 MHz, $CDCl_3$).

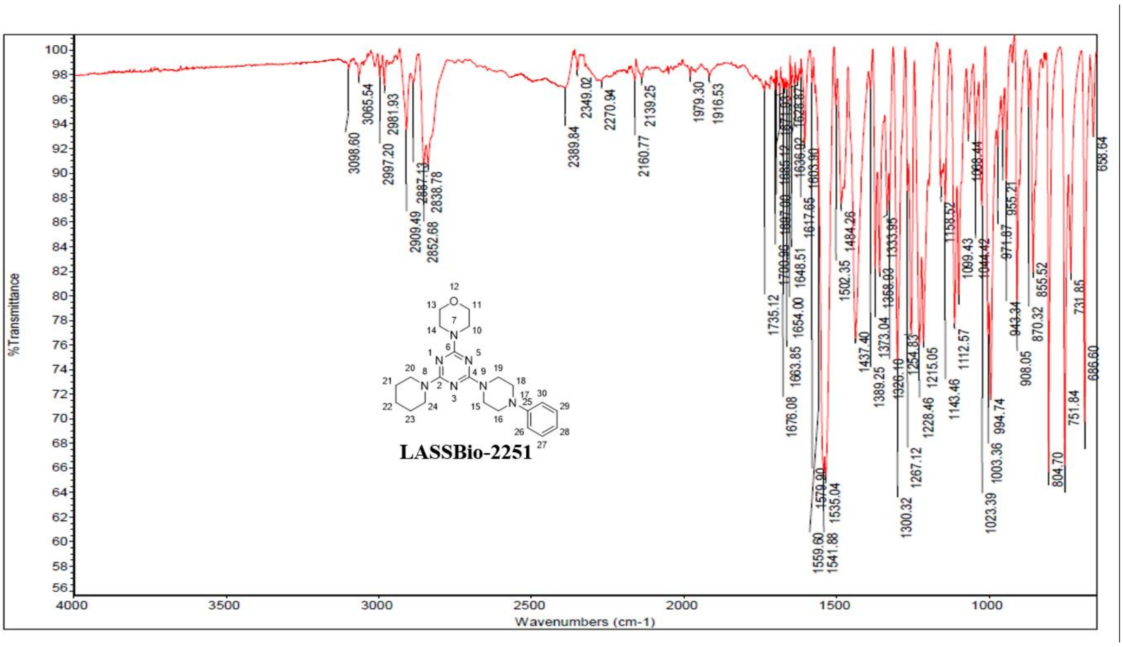

Figure S15. Absorption spectra in the infrared region of 4-(4-(4-phenylpiperazin-1-yl)-6-(piperidin-1-yl)-1,3,5-triazin-2-yl)morpholine (**5b**) (ATR).

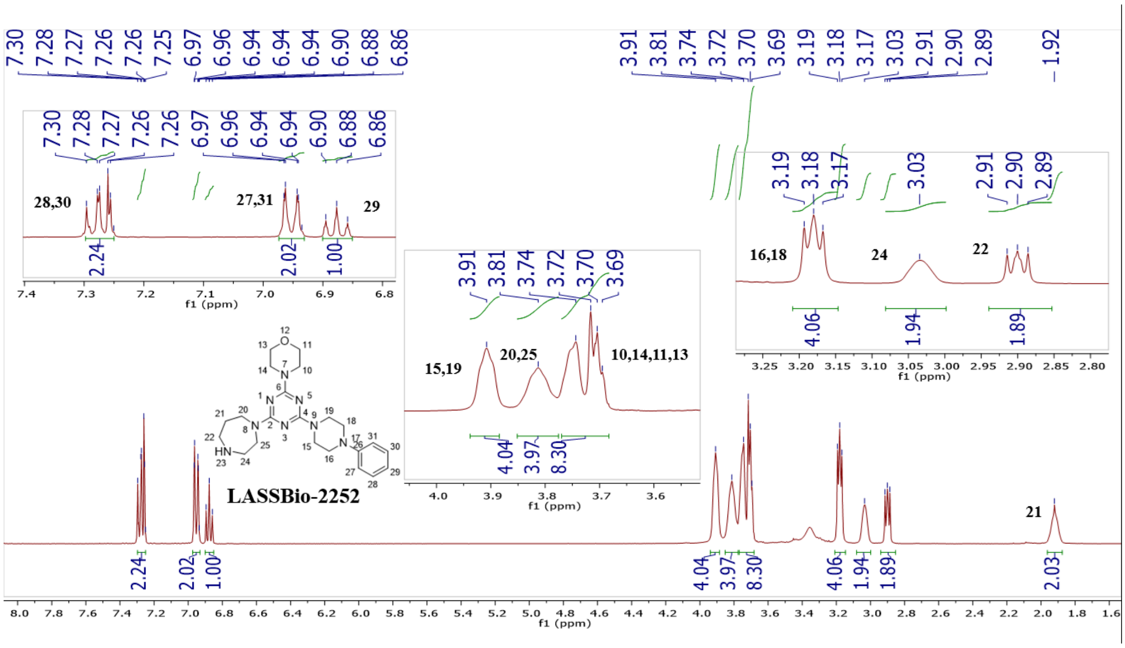

Figure S16. ^{1}H NMR spectra of 4-(4-(1,4-diazepan-1-yl)-6-(4-phenylpiperazin-1-yl)-1,3,5-triazin-2-yl)morpholine (**5f**) (400 MHz, DMSO-d_6,).

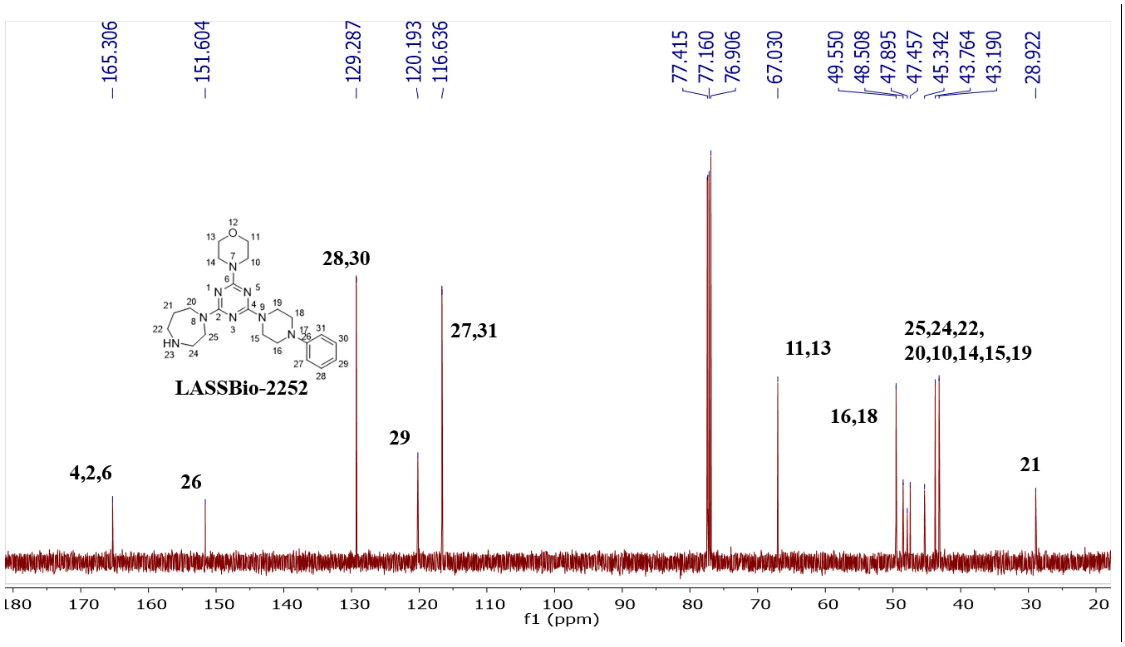

Figure S17. ^{13}C NMR spectra of 4-(4-(1,4-diazepan-1-yl)-6-(4-phenylpiperazin-1-yl)-1,3,5-triazin-2-yl)morpholine (**5f**) (400 MHz, $CDCl_3$).

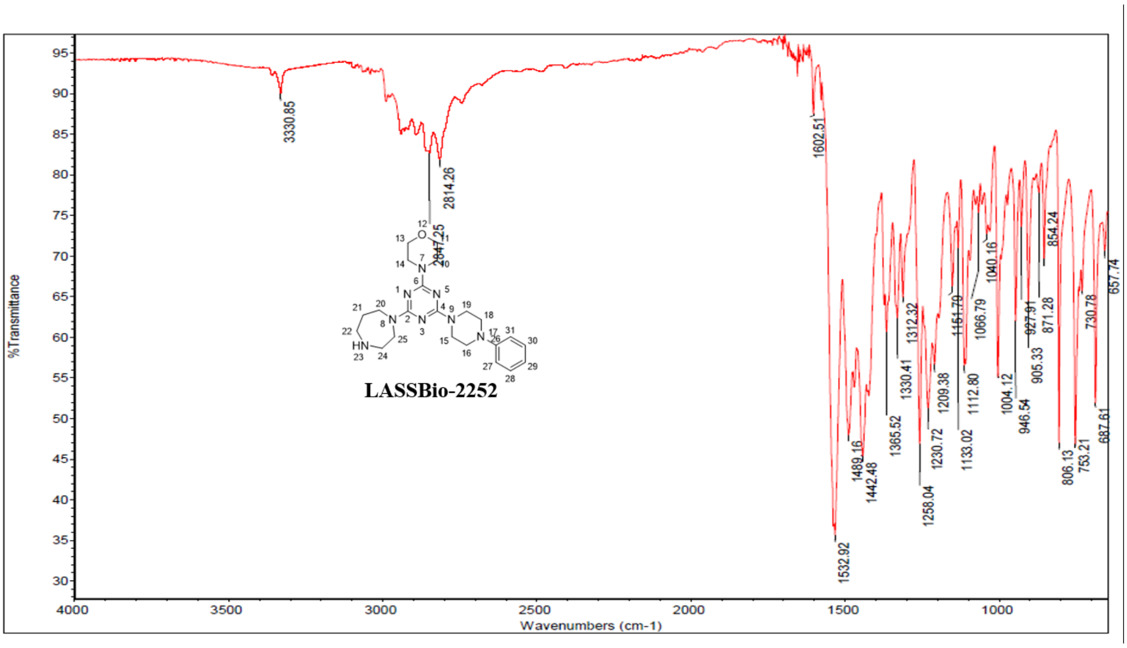

Figure S18. Absorption spectra in the infrared region of 4-(4-(1,4-diazepan-1-yl)-6-(4-phenylpiperazin-1-yl)-1,3,5-triazin-2-yl)morpholine (**5f**) (ATR).

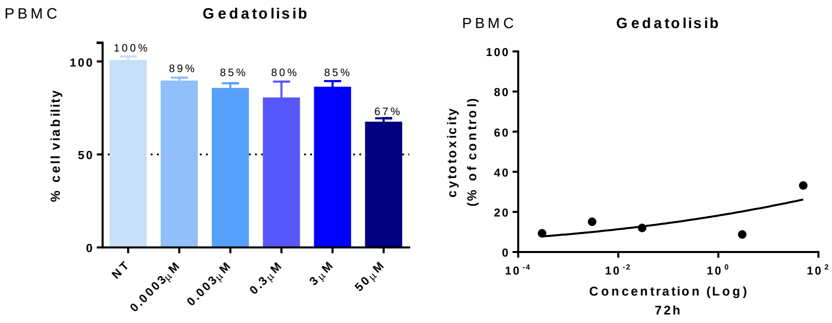

Figure S19. Cytotoxicity graph and curve as a function of Gedatolisib **(4)** concentration variation **(01)** against Peripheral Blood Mononuclear Cells, from MTT assay in 72 hours. Data presented as mean ± standard error of the mean of three independent experiments.

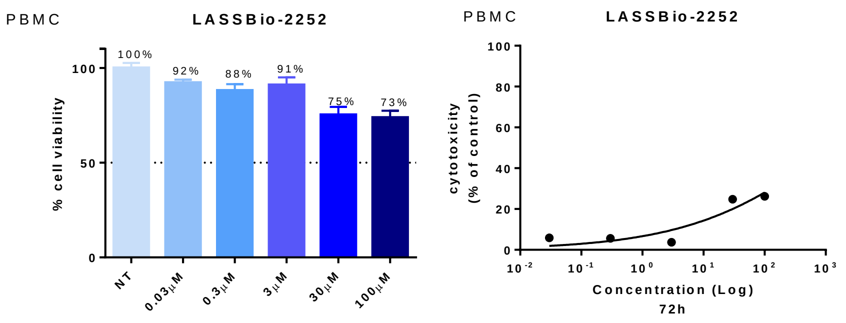

Figure S20. Cytotoxicity graph and curve as a function of LASSBio-2252 (**5f**) concentration variation against Peripheral Blood Mononuclear Cells, from MTT assay at 72 hours. Data presented as mean ± standard error of the mean of three independent experiments.
